# Oral health of Dutch primary school children in relation to social and health aspects

**DOI:** 10.2340/aos.v83.42053

**Published:** 2024-10-03

**Authors:** Brenda G. Grift, Denise Duijster, Geert J.M.G. van der Heijden, Josef J.J.M. Bruers, Katarina Jerković-Ćosić

**Affiliations:** a Utrecht University of Applied Sciences, Research Group Innovation in Preventive Healthcare, Utrecht, The Netherlands; b Academic Centre for Dentistry Amsterdam (ACTA), Department of Oral Public Health, University of Amsterdam and VU University, Amsterdam, The Netherlands

**Keywords:** Oral health, inequality, increased risk, health status, life style

## Abstract

**Objective:**

This research investigated the oral health status of Dutch primary school children aged 10–12 years in Utrecht and its association with demographic characteristics, lifestyle factors, general psychosocial health, and dental treatment, to guide targeted interventions to improve children’s oral and overall well-being.

**Materials and methods:**

A cross-sectional survey was conducted at 49 primary schools in Utrecht in 2017 and 42 schools in 2019. The questionnaire covered background characteristics (7 questions), psychosocial health (14 questions), nutrition and exercise (20 questions), school and leisure time (26 questions), home situation (23 questions), and oral health (4 questions). Associations were analyzed using multivariate logistic regression.

**Results:**

Data from 5,426 children were analyzed. Prior to the survey, 11% did not visit a dentist, 23% had a toothache, 22% had a cavity filled, and 8% had a tooth extracted. Independent predictors for increased odds of oral health problems were migration background, poor general health, drinking more than two glasses of soft drinks per day, having ever drunk alcohol, having fear of failure, experiencing problems at home and/or coming from average or low socioeconomic position group. Factors associated with increased odds of dental treatment, such as cavity filled and tooth extracted, were migration background, fear of failure and home problems.

**Conclusions:**

These findings emphasize the need for targeted prevention to improve the oral health of children with a migration background, average or low socioeconomic position and/or with poorer general and psychosocial health, unhealthy diets and problems in the home situation, within a community-based and transdisciplinary approach.

## Introduction

Health is defined as the ability to adapt and take control against physical, emotional and social challenges in life [1]. According to the FDI World Dental Federation, the definition of oral health is also multifaceted and includes the ability to speak, smell, taste, touch, chew, swallow, and convey a range of emotions through facial expression, without pain, discomfort or disease [2]. Furthermore, the FDI states that oral health is a fundamental part of physical and mental well-being.

Oral health and oral health care of all people require permanent attention from professionals and policymakers. This especially applies to the oral health of children from the eruption of the first tooth [3]. Oral health behaviors, such as toothbrushing twice daily, are often shaped early in life and dental caries experience in childhood is a strong predictor of oral health problems in adult life [4]. In 2017, in the Netherlands 29% of the 5-year-olds, 43% of the 11-year-olds and 65% of the 17-year-olds had dental caries [5]. Dental caries can be prevented by twice-daily toothbrushing with fluoride toothpaste and by limiting sugary foods and drinks [6, 7]. In addition, research has shown that oral health and general health often share risk factors related to health behavior and underlying (psycho)social influences [8, 9]. Health behaviors of children and young adolescents such as diet, physical activity, tobacco and alcohol use, have been shown to be associated with factors such as gender, age, peer pressure, stress, parents’ educational background, ethnic group and school environment [8, 10–12]. It is therefore a preferable choice to apply an integrated approach, such as community-based health promotion program, including oral health. This method simultaneously improves general and oral health by addressing common risk factors and promoting healthy behaviors. An example in the Netherlands is the ‘Jongeren op Gezond Gewicht’ (JOGG) program [13]. This program promotes an integrated approach by working with local communities to create environments that encourage healthy living. The JOGG initiative integrates oral health education with broader health promotion activities, such as encouraging healthy eating, physical activity, and reducing sugary drink consumption. This holistic strategy helps improve both general health outcomes for children and adolescents [13, 14]. Such an approach to health promotion is more cost-effective than focusing on a single disease. According to Petersen [15], it ‘avoids duplication of efforts, inefficient use of resources or even worse, conflicting messages’.

To develop an effective integrated approach for addressing both general and oral health in the Netherlands, it is crucial to identify the factors associated with oral health. Understanding these factors is essential for creating targeted interventions. In response to this need, the municipality of Utrecht has established the Youth Monitor Utrecht (YMU). This program biannually charts the health of primary school children aged 10–12 years [13]. Utrecht, the fourth largest city in the Netherlands, has a diverse population in terms of socioeconomic characteristics and migration background. The YMU covers various health and sociodemographic topics, and as of 2017, it includes four questions about oral health (care). The results of this monitor can provide valuable insights into the association between general and psychosocial health on one side and oral health and dental care utilization on the other, which are crucial for developing an integrated approach to improve oral and general health in children. This initiative underscores the importance of comprehensive data collection and analysis to inform health policies and improve outcomes for the community.

Therefore, the aim of this research was to study (1) the oral health status of primary school children in Utrecht, The Netherlands; (2) the association of children’s oral health with demographic characteristics, lifestyle factors, and general psychosocial health; and (3) factors associated with whether children have had dental treatment in the past.

## Methods

This study was based on two cross-sectional monitoring surveys as part of the YMU, carried out in October–November 2017 and October–November 2019. The data from both years were collected using the same method for sampling and data collection, which made it possible to aggregate the data and analyze them as one dataset.

### Study sample

The study population included primary school children aged 10–12 years in the municipality of Utrecht, the Netherlands. The municipality is divided into 10 districts and approximately four schools per district were recruited. Participation of at least 100 primary school children per district is required for reliability. Regarding the representativeness of the study, the distribution of schools across the neighborhood was considered during the selection process. More schools from disadvantaged areas, characterized by significant health disparities, were recruited to participate. In Utrecht, the neighborhoods of Overvecht and Zuid-West experience significant health disparities, with higher rates of health issues and poorer quality of life compared to other parts of the city [16]. This approach by YMU aimed to provide reliable sub-neighborhood data and ensure the accurate representation and addressing of community needs. Schools that participated in the previous monitor were approached first. If a school declined, a replacement within the same district with a similar educational philosophy was selected.

In 2017, 49 schools participated and 10 schools declined participation. In 2019, 13 schools declined participation and 6 new schools were included. As a result, 42 schools ultimately participated in the study in 2019. All children in the final two grades of the selected primary schools were invited to participate.

### Study tool

A questionnaire designed by the municipality of Utrecht was used for data collection. The questionnaire consisted of 94 questions on demographic characteristics (*n* = 7), health-related topics, including general health (*n* = 7), psychosocial health (*n* = 7), nutrition and exercise (*n* = 20), leisure time (*n* = 9), school (*n* = 17) home situation (*n* = 23), and oral health (*n* = 4).

#### Dependent variables

The dependent variables on oral health were measured by four dichotomous questions: whether the child had a dental check-up; toothache; restorative treatment of caries and extraction of teeth caused by caries or toothache, all in the past year. The items used are presented in Table 1.

**Table 1:** Variables included in this study.

Dependent variables	Coding	Recoding
Oral health		
• Dental check-up in the past year	Yes *or* no	
• Toothache in the past year	Yes *or* no	
• Cavity filled in the past year	Yes *or* no	
• Tooth extracted in the past year	Yes *or* no	
**Independent variables**	**Coding**	**Recoding**
Demographic characteristics		
• Gender	Boy *or* girl	
• Grade	Grade 10 *or* grade 11	
• Migration background	Dutch, Western *or* non-Western	
General health	Very good, good, going well, moderate *or bad*	Good (very good + good) Not good (going well + moderate + bad)
Nutrition		
• Days per week breakfast		Daily (≥ 5 days p/w)
		Not daily (<4 days p/w)
• Days per week fruits	(almost) never, 1 day p/w, 2 days p/w, 3 p/w, 4 days p/w, 5 days p/w, 6 days p/w *or* every day	Daily (every day)
		Not daily (almost never – 6 days p/w)
• Days per week soda, juice etc.		Daily (every day)
		Not daily (almost never – 6 days p/w)
		
• Number of glasses per day soda, juice etc.	1 glass p/d, 2 glasses p/d, 3 glasses p/d, 4 or more	< 2 glasses p/d
	glasses p/d	≥ 2 glasses p/d
Smoking and alcohol		
• Ever smoked	Yes *or no*	No (never)
• Ever drank alcohol	Never, 1 or a few sips, occasionally but not every week, weekly	Yes (1 or a few sips + occasionally + weekly)
• Drinking alcohol at home	I am allowed and I do it, I am allowed but I don’t do it, it’s not allowed and I’m not doing it, it’s not allowed but I’m doing it	I am allowed (I do it + I am not doing it)
		I am not allowed (I don’t do it + I do it)
Psychosocial health		
• Low self-confidence	Yes *or* no	
• Fear of failure	Yes *or* no	
• Total score psychosocial health (strengths and difficulties questionnaire (SDQ)) [24]		
• Emotional problems	For all six scales	Not at risk (normal)
• Behavioral problems	Normal, at risk, increased	At risk (At risk + increased)
• Hyperactivity		
• Problems with peers		
• Pro-social behavior problems		
Leisure time		
• Physical activity (Dutch standard of healthy exercise (NNGB))	Inactive, semi-inactive, semi-active, norm-active	Inactive (inactive + semi-inactive + semi-active)
		Norm-active
• Screentime per day	Less than half an hour a day, half an hour to 1 hour a day, 1–2 h a day, 2–3 h a day, 3–6 h a day, 6 h or more per day	< 2 h per day
		≥ 2 h per day
Family circumstances		
• Family composition	With father and mother *or* else	
• Relationship with parents	Below average, average, above average	Lower than average
		Average and above
• Problems at home	Yes *or* no	
• Family member has illness, disability or addiction	Yes *or* no	
• Extra tasks at home	Yes *or* no	
• Social Economic Position (Family Affluence Scale (FAS)) [25]	Low, average *or* high	

#### Independent variables

This study focused on the factors that may be related to the children’s oral health. Therefore, not all variables of the questionnaire are covered in this study. Based on previous literature regarding possible factors affecting oral health, a selection of independent variables was made for this study: demographic characteristics (i.e. migration background), general health status, dietary habits, lifestyle habits such as smoking, use of alcohol, physical activity and screen time, psychosocial health and home situation [17] [18, 20]. According to the questionnaire, home situation includes the relationship the child has with their parents, problems experienced with parents, whether the child has to provide extra care within the family, performing extra tasks such as household chores and the economic position of the family. The socioeconomic position (SEP) is determined using the six questions of the Family Affluence Scale (FAS III)[20, 21]. A total of 13 points can be scored on the 6 questions. A score of 0–7 indicates low SEP, 8–11 medium SEP and 12–13 high SEP [22]. Children were classified with a migration background if one of the parents was born in a country other than the Netherlands. The items used in the analyses are presented in Table 1.

Within psychosocial health, the underlying components were self-confidence, fear of failure, emotional problems, behavioral problems, hyperactivity, problems with peers, and pro-social behavior. Fear of failure refers to the anxiety or apprehension individuals feel about not meeting their goals or expectations. It is characterized by concerns about future outcomes and personal performance. Based on the youth monitor questionnaire, it involves various aspects such as: worrying about the future, fear of not succeeding, concerns about accomplishing tasks and fear of making mistakes. The Strengths and Difficulties Questionnaire (SDQ) was used to submit questions about emotional problems, behavioral problems, hyperactivity, problems with peers, and pro-social behavior. Within family circumstances, the underlying components were the relationship with parents and socio-economic position. Table 2 presents the Cronbach’s alpha values of these underlying components, provided by researchers from the municipality of Utrecht. A score above 0.70 signifies internal consistency. The underlying components of psychosocial health measured using the SDQ had low internal consistency and were not included in the analysis. Only the total SDQ score was used.

**Table 2:** Cronbach’s alpha of the underlying subconstructs.

Sub constructs	Cronbach’s alpha	Number of items (*n*)	Internal consistency
Psychosocial Health			
Self-confidence	0.818	3	
Fear of failure	0.743	3	
SDQ Total	0.749	25	
SDQ: emotional problems	0.664	5	*Not reliable*
SDQ: behavioral problems	0.295	5	*Not reliable*
SDQ: hyperactivity	0.644	5	*Not reliable*
SDQ: problems with peers	0.332	5	*Not reliable*
SDQ: pro-social behavior	0.517	5	*Not reliable*
Family circumstances			
Relationship with parents	0.812	6	
Social Economic Position (Family Affluence Scale)[25]	0.475	6	Low internal consistency, but validated instrument

### Data collection

Prior to data collection, parents received a letter from the school detailing the questionnaire’s content, purpose, and the subsequent use of the data. They were given the opportunity to object to their child’s participation by filling out a form or sending an email to the child’s teacher. The questionnaire was taken anonymously and on paper by children themselves in their classroom. A trained research assistant from the municipality supervised the procedure, supported children who had difficulty answering the questions, and collected the questionnaires. Teachers were present but did not actively participate. The administration of the questionnaire lasted approximately 1 h. The questionnaires were processed automatically, entering the data based on the checkboxes children filled in.

### Data analysis

Statistical analyses were performed using SPSS version 27 [25]. To assess the oral health status of primary school children in Utrecht, descriptive statistics were performed using the four oral health variables.

To examine the association of demographic characteristics, lifestyle factors, general and psychosocial health with children’s oral health, children were categorized into two groups based on their oral health status:

- Children who are monitored: These are children who have been to the dentist for a check-up in the past year and who have not had a toothache. Not visiting a dentist has been reported to be associated with poor oral health and toothache has been reported to result from not visiting a dental care professional in time [26, 27].
- Children who are at risk: These are children who have not been to the dentist for a check-up in the past year, or who have had a check-up but had a toothache. According to Xu et al., children visit the dentist more if they experience dental pain [28]. Less preventive dental visits were also associated with a higher number of decayed, missing and filled teeth (dmft). Therefore, children who either do not visit a dentist for check-up or suffer from toothache were classified as at risk for oral health problems.

Based on these two groups, an univariate logistic regression analysis was performed to select factors that should be included in the multivariate analysis. Subsequently, a multivariable logistic regression analysis was performed using the independent variables that were significantly related to oral health status in the univariate analysis (*p* < 0.05). A stepwise backward procedure was used. Only the independent variables that remained significantly associated with oral health status were shown in the final model. The level of significance was established at 5% (α < 0.05).

To examine which factors were associated with whether children have had dental treatment in the past year, a logistic regression was performed using the dependent variable ‘dental treatment’ (cavity filled and/or tooth extracted in the past year). The analysis was adjusted for oral health status (i.e. monitored or at risk) as a covariate.

## Results

In 2017, 2,684 children from 49 schools completed the survey. In 2019, 2,740 children from 42 schools completed the survey. A total of 5,424 children aged 10–12 years were included in this study (*n* = 2.359 ≤10 years, *n* = 3,018 ≥11 years old). On average, in 2017 and 2019, 92% of all invited children completed the questionnaire. In 4% of the children, the parents of the children refused to participate, 3% of the children were sick and 0.5% had other reasons. The distribution of boys (49%) and girls (51%) in the study sample was almost even. Of all the children, 40% had a migration background of which three-quarters was non-Western. The majority of all children (84%) lived with both parents and more than half of the children (53%) came from a family with a high SEP.

### Oral healthcare of primary school children in Utrecht

Data on oral healthcare is presented in Table 3. In 2017, 1 in 7 children (13%) did not visit the dentist for a check-up in the year prior to the survey. This percentage was lower in 2019, when 9% of the children had not been to the dentist for a check-up. In addition, of the total study sample, 1 in 5 children had suffered from toothache (23%) or had at least one cavity filled (22%). Of the total number of children, almost 1 in 10 (8%) had a tooth extracted.

**Table 3:** Oral health and oral healthcare of children in Utrecht in 2017 and 2019.

Oral health variables*	Year 2017 (*n* = 2.686)	Year 2019 (*n* = 2.740)	Total (*n* = 5.426)
	*n* (%)	*n* (%)	*n* (%)
Dental check-up in the past year			
Yes	2,276 (85)	2,686 (91)	4,761 (88)
No	353 (13)	230 (8)	583 (11)
Toothache in the past year			
No	2,071 (77)	2,077 (76)	4,148 (76)
Yes	602 (22)	642 (23)	1,244 (23)
Cavity filled in the past year			
No	2,106 (78)	2,120 (77)	4,226 (78)
Yes	567 (21)	596 (22)	1,163 (21)
Tooth extracted in the past year			
No	2,443 (91)	2,499 (91)	4,942 (92)
Yes	213 (8)	213 (8)	442 (8)
Oral health status			
Monitored	1,756 (65)	1,889 (69)	3,645 (67)
At risk	860 (32)	812 (30)	1,672 (31)

* Approximately 1%–2% of the oral health questions were left unanswered.

### Oral health status in relation to demographic characteristics, lifestyle factors, and general psychosocial health

According to the categorization of the two groups based on oral health status (monitored versus at risk), 31% of the children were at risk of oral health problems. The multivariate analysis (Table 4) found that greater odds of being at risk of oral health problems were observed among children with a non-Western background (OR: 1.3 [95% confidence interval = 1.1;1.5]), with poor general health (OR: 1.4 [1.1;1.8]), who drank more than two soft drinks or juices per day (OR: 1.3 [1.2;1.5]) or ever drunk alcohol (OR: 1.4 [1.2;1.7]). In addition, children with fear of failure (OR: 1.4 [1.2;1.7]), who experienced problems at home (OR: 1.4 [1.2;1.7]) and children from an average (OR: 1.2 [1.1;1.4]) or low (OR: 1.7 [1.3;2.2]) SEP were at significantly higher risk of poor oral health.

**Table 4:** Demographic characteristics, lifestyle factors and general psychosocial health factors associated with oral health status of children.

Independent variables (*N* = 5,426)	*OR (CI) univariate*	*OR (CI) multivariate*	*p*
Children who are monitored^a^ (ref) *versus* children who are at risk^b^			
*Demographic characteristics*			
Migration background			
• None (*n* = 3,229)	Ref	Ref	Ref
• Non-Western^c^ (*n* = 1,728)	1.5 (1.3 – 1.7)	1.3 (1.1 – 1.5)	0.004
• Western^c^ (*n* = 469)	1.1 (0.9 – 1.4)	1.1 (0.9 – 1.5)	0.331
*Dental treatment*			
Tooth extracted^e^ (*n* = 442)	4.5 (3.6 – 5.5)	3.8 (2.9 – 4.8)	< 0.001
Cavity filled^e^ (*n* = 1,163)	1.7 (1.5 – 2.0)	1.2 (1.0 – 1.4)	0.022
*General health*			
Poor general health^e^ (*n* = 495)	2.0 (1.6 – 2.4)	1.4 (1.1 – 1.8)	0.004
*Nutrition*			
> 2 glasses soda or juice per day^e^ (*n* = 1,830)	1.5 (1.3 – 1.7)	1.3 (1.2 – 1.5)	< 0.001
*Smoking and alcohol use*			
Ever drank alcohol^e^ (*n* = 929)	1.3 (1.1 – 1.5)	1.4 (1.2 – 1.7)	< 0.001
*Psychosocial health*			
** **Fear of failure^e^ (*n* = 739)	1.8 (1.6 – 2.1)	1.4 (1.2 – 1.7)	< 0.001
*Family circumstances*			
Problems at home^e^ (*n* = 1.038)	1.6 (1.3 – 1.8)	1.4 (1.2 – 1.7)	< 0.001
Social economic position			
• High (*n* = 2,724)	Ref	Ref	Ref
• Average (*n* = 2,027)	1.3 (1.1 – 1.5)	1.2 (1.1 – 1.4)	0.004
• Low (*n* = 390)	2.0 (1.6 – 2.5)	1.7 (1.3 – 2.2)	< 0.001

a Children with dental visit and no toothache.

b Children with no dental visit OR dental visit with toothache.

c Non-Western countries include Africa, Latin America, Asia (excluding Indonesia and Japan), and Turkey.

d Western countries include Europe (excluding Turkey), North America, Oceania, Indonesia, and Japan.

e Dummy variable (1/0).

### Factors associated with dental treatment

Within this study, 1,605 (30%) children have had dental treatment in the past year. Children classified as at risk of oral health problems had significantly more cavities filled and teeth extracted in the past year compared to children who were monitored (*p* < 0.001). The multivariate analysis (Table 5) shows greater odds of having dental treatment in the past year among children with a non-Western migration background (OR: 2.1 [1.7;2.6]), children who drink more than two soft drinks or juices per day (OR: 1.4 [1.1;1.7]), who don’t eat breakfast daily (OR: 1.6 [1.1;2.3]), who have fear of failure (OR: 1.5 [1.1;2.0]) and who have extra tasks at home (OR: 1.3 [1.1;1.6]).

**Table 5:** Demographic characteristics, lifestyle factors and general psychosocial health factors associated with dental treatments.

Independent variables *n* = 5,426	*OR (CI) univariate*	*OR (CI) multivariate*	*p*
No dental treatment (ref) *versus* dental treatments^a^			
*Demographic characteristics*			
Migration background			
• None (*n* = 3,229)	Ref	Ref	Ref
• Non-Western (*n* = 1,728)	2.5 (2.2–2.8)	2.1 (1.7–2.6)	< 0.001
• Western (*n* = 469)	1.3 (1.0–1.6)	1.3 (0.9–1.9)	0.147
*Nutrition*			
> 2 glasses soda or juice per day^b^ (*n* = 1,830)	1.5 (1.3–1.7)	1.4 (1.1–1.7)	0.003
Not eating breakfast everyday^b^ (*n* = 353)	2.4 (1.9–3.0)	1.6 (1.1–2.3)	0.011
*Psychosocial health*			
Fear of failure^b^ (*n* = 739)	1.6 (1.3–1.8)	1.5 (1.1–2.0)	0.004
*Family circumstances*			
Extra tasks at home^b^ (*n* = 1,021)	1.6 (1.3–1.9)	1.3 (1.1–1.6)	0.014

a Adjusted for dental treatment.

b Dummy variable (1/0).

## Discussion

The findings show that 1 in 3 (31%) children in the age range of 10 to 12 years in the municipality of Utrecht were at risk for oral health problems. Within this study population, 1 in 10 (11%) children did not visit the dentist in the past year for a check-up and 30% had dental treatment in the past. Factors associated with higher risk of oral health problems in children are migration background, average or low SEP, poor general health, poor psychosocial health, unhealthy diets and problems in the home situation. Similar associations were observed with dental treatment, including tooth restorations and tooth extractions.

The results indicating that a risk of poor oral health was significantly higher in children with a non-Western background, average to low SEP, poor general health, an unhealthy diet, are not that surprising and support previous studies [17, 18, 29]. Similar research has been conducted in Finland by the Finnish National School Health Promotion Study [30], and Denmark [31] indicating that socio- and health aspects are associated with oral health behavior. Similar to the Netherlands, Denmark and Finland provide free public dental services for children up to 18 years, yet inequality in oral health persists. Inequalities established in childhood persisted throughout the whole life course [32]. However, the relationships between oral health and poor psychosocial health, or stressful family circumstances are less studied. In our study, we found a significant association between poor oral health and poor psychosocial health in children. Additionally, our finding indicates an association between stressful family environments and poor oral health. This shows that oral health problems are a multi-factor phenomenon and are not only related to general health factors, but also well-being and living circumstances indicating the need to include oral health promotion within the broad programs aimed at healthy living and upbringings. In order to promote oral health, it is therefore important to take behavioral determinants and sociodemographic factors into account.

One of the main contributions of this study is the identified association between factors related to dental check-ups aimed at monitoring of dental health and offering oral health promotion by professionals. Dental caries is a preventable disease, and therefore dental treatment is preventable as well [33]. It is important that a dentist is visited regularly for check-ups and oral health promotion [34] However, in this study population, 1 in 10 children did not visit the dentist for a check-up. Timely monitoring of dental health and the oral health promotion offered by professionals are essential for preventing and addressing oral health issues effectively. According to Reda et al. [35], not visiting a dentist for dental check-ups is associated with poorer general health and poorer oral health. This is also reflected in the study by Xu et al. [28]. They indicate that children who do not regularly attend dental check-ups visit the dentist more frequently due to toothaches or dental decay [28]. In addition, children with a migration background who use tobacco and drink soda not only have poorer oral health but also visit the dentist for check-ups less frequently, according to Chertok et al. [34]. Previous research also shows that large inequalities have been identified in oral healthcare service utilization in children [28] especially socioeconomic differences, whereas children from a low SEP visit the dentist less regularly for check-ups [35]. Low parental oral health literacy and parental language proficiency, which are prevalent among people with a low SEP and/or migration background, contribute to health inequalities[36]. Opportunities exist not only for dental professionals to address this problem but also for youth healthcare professionals who visit primary schools. They could ask children if they attend dental check-ups and, if not, recommend that they do so.

The toothache prevalence in this study was slightly more favorable than the findings of a recently published review showing that 4 in 10 children worldwide in the age of 6–12-year old suffered from toothache [37], which is mostly related to dental caries [38]. Caries significantly affects children’s quality of life. It causes chewing difficulties and changes in eating patterns, low self-esteem, social problems, and poorer school performance. This decline can create a vicious cycle for children from low socioeconomic backgrounds. Poor oral health leads to more absenteeism and lower academic achievement, which in turn limits future opportunities [39–41]. In addition to the effect on the overall well-being of the child, the economic burden of dental caries treatment is high. Treatment costs due to dental diseases correspond to an average of 4.6% of global health expenditure[42]. In the Netherlands, the costs for treating caries exceeded 448 million euros in 2022 [43]. Improving the oral health of children can lead to better general health but also economic benefits.

The oral health professional plays a crucial role in monitoring and preventing oral diseases. However, individual prevention in oral healthcare practices primarily focuses on promoting healthy oral health behaviors. Other important aspects of children’s general health and well-being are addressed within public health. Youth Public Healthcare also plays a significant role in monitoring oral health but often lacks the time and knowledge to do so effectively [44]. Therefore, it is essential to acknowledge the collaboration between youth healthcare and oral health professionals in ensuring comprehensive oral health care throughout different stages of childhood development. The Ministry of Health, Welfare, and Sport (VWS), in collaboration with various parties from dental care and public health, is exploring potential opportunities to improve the oral health of young people and determining the steps necessary to achieve this improvement [45].

Some limitations should be kept in mind when interpreting the findings of this study. First, data for this study was acquired through a survey, as part of the YMU. The questionnaire itself was developed by the Public Health Department of the municipality Utrecht, the Netherlands. It is crucial to highlight that the researchers played no role in determining the specific questions included in the questionnaire. Second, this study is based on self-reports of children between the ages of 10 and 12 years. It is very likely that children of this age do not always give accurate accounts of their behavior, lifestyle, habits and experiences. For example, children lack a good sense of time, and certainly the memory of things such as toothache or tooth extraction can linger for a long time. Therefore, the report of when it happened is not always reliable. Also, the questions about which dental care services were performed were not included in the questionnaire. Third, the underlying subconstructs of the SDQ had low internal consistency and were therefore not included in the analysis. In this study, only the total SDQ score was considered. As a result, it is not clear exactly which factors related to the SDQ are associated with oral health. However, separate questions about self-confidence and fear of failure were included in the questionnaire, which enables statements related to psychosocial health. This also applies for the total score of the SDQ. This is a very useful questionnaire to measure psychosocial health and is widely used nationally and internationally [23, 46, 47]. Fourth, the nature of the associations that have been found is unknown, as this study was not designed to investigate causal relationships. It is therefore uncertain what the nature of relationship is with for example SES or migration background, only that these factors are associated to oral health. Lastly, the socioeconomic status of the study population is high compared to the rest of the Netherlands. However, Utrecht has a diverse population in terms of socioeconomic characteristics and migration background. In addition, extra schools in disadvantaged were included by the municipality to make the results more generalizable to the rest of the Netherlands.

Despite these limitations, a strength of this study is the large population sample. In 2019, approximately 2,800 children at 42 schools completed the questionnaire. That is 38% of the approximately 7,300 children of the final two grades in the primary schools in Utrecht. There is a possibility that some children may have redone the same schoolyear and filled in the questionnaire twice in 2017 and 2019, but these will only be a very few. The size and the diversity (e.g. children from all districts were included) of the study population contribute to the internal validity of this study.

The youth monitor is carried out every 2 years, enabling long-term monitoring at the district level, which enables the municipality to deploy targeted interventions per district.

The findings of this study indicate great importance for assessing relations between oral health and other aspects of general and psychosocial health in schoolchildren. This study shows that poor oral health can be related to multiple problems, such as fear of failure. These aspects regarding psychosocial health are still slightly underexposed in the literature on oral health. Most studies on association with oral health in children focus primarily on demographic characteristics, oral health behavior and on the psychosocial factors of the parents[48, 49]. In addition, the focus is often on the influence of poor oral health on the quality of life of children[50, 51]. Therefore, in order to gain knowledge on important aspects that should be addressed in oral health prevention program, further research should include data on psychosocial health of children, such as emotional problems, behavioral problems, hyperactivity, problems with peers and pro-social behavior problems. Further research should aim on children from a problematic home situation and the association with oral health. This study shows that psychosocial factors are associated with a risk of poor oral health, but how this is related and which psychosocial factors are relevant remains unclear. An efficient approach to gathering these data would be to integrate oral health questions into existing health cohorts and monitoring programs. Additionally, conducting qualitative studies would provide deeper insights into how and why psychosocial factors influence oral health. By incorporating qualitative approaches, such as interviews or focus groups, alongside quantitative data collection methods, a more comprehensive understanding of these associations can be achieved. This mixed-methods approach would provide deeper insights into the lived experiences, perceptions, and contextual factors influencing oral health outcomes in vulnerable populations.

In conclusion, this study found that 1 in 3 children in the age group of 10–12-years were at risk of oral health problems. Many children do not visit the dentist regularly for a check-up and/or have had dental treatment because of dental caries or toothache. Poor oral health is associated with many sociodemographic and behavioral aspects, such as migration background, unhealthy diet, and poor psychosocial health. To enhance children’s oral and overall health, an integrated public health approach that promotes healthy behaviors and adapts to the home environment is recommended. Health care and welfare sector should be involved, but also in involving children and their parents in the development of an integrated approach is important for its success. Their perspectives and needs are invaluable in shaping effective strategies that address oral health issues. By including them, interventions will be tailored to meet specific needs, resulting in more impactful results. Attention should be devoted to promoting healthy eating and drinking habits among children through health education, community interventions and creating supportive school, but also on healthy living circumstances. In the Netherlands, there is an opportunity to include oral health in community programs such as JOGG [13] and Healthy Schools [52].

## Data Availability

This study was carried out in collaboration with the municipality of Utrecht, the Netherlands. Data were obtained through the public health monitor by the municipality and shared with the researchers.
